# Course of Testosterone Levels and Potential Biochemical Biomarkers of Androgenization with a Conservative Protocol of Androgen Hormone Therapy in Transgender Males

**DOI:** 10.3390/jcm15166498

**Published:** 2026-08-21

**Authors:** Guillermo Velasco de Cos, María Teresa García Unzueta, Ana Moyano Martínez, Pedro Muñoz Cacho, Aurelia Villar Bonet, Armando Raul Guerra Ruiz, Bernardo A. Lavin-Gómez, Maialen Ormazabal Monterrubio, Luis Alberto Vázquez

**Affiliations:** 1 Clinical Laboratory, Valladolid University Hospital, 47003 Valladolid, Spain; gvelascod@saludcastillayleon.es; 2 Biochemistry and Clinical Analysis Department, Marqués de Valdecilla University Hospital, 39008 Santander, Spain; armandoraul.guerra@scsalud.es (A.R.G.R.); bernardoalio.lavin@scsalud.es (B.A.L.-G.); maialen.ormazabal.m@gmail.com (M.O.M.); 3 Faculty of Medicine, University of Cantabria, 39005 Santander, Spain; luisalberto.vazquez@scsalud.es; 4 Institute for Research Marqués de Valdecilla (IDIVAL), 39011 Santander, Spain; 5 Clinical Laboratory, Salamanca University Healthcare Complex, 37007 Salamanca, Spain; amoyano@saludcastillayleon.es; 6 Department of Medicina Familiar y Comunitaria (IDIVAL), 39008 Santander, Spain; pedro.munoz@scsalud.es; 7 Endocrinology and Nutrition, 39008 Santander, Spain; avb666@hotmail.com; 8 Department of Endocrinology, Diabetes and Nutrition, University Hospital Marqués de Valdecilla, 39008 Santander, Spain

**Keywords:** transgender males, gender-affirming hormone therapy, testosterone therapy, safety, sex steroids, hematocrit, lipids, prostate-specific antigen

## Abstract

**Objectives:** This study aimed to evaluate the hormonal, hematologic, and biochemical outcomes of a conservative protocol for androgen therapy for transgender males. **Methods**: This was an observational, prospective, and comparative study conducted in the gender identity clinic of a tertiary university hospital. Testosterone therapy consisted of testosterone cypionate administered intramuscularly every month at an initial dosage of 25 mg/month, which was increased to 50 mg/month at month 3, 100 mg/month at month 6, and 250 mg/month at month 9. Assessments consisted of a complete blood count, biochemical tests, urine analysis, and hormonal tests. We also administered the Transgender Congruence Scale (TCS) post hoc. **Results**: We recruited 40 transgender males with a mean (SD) age of 22.5 (8.4) years. Only changes in the hematocrit were statistically significant as early as month 9. There were no significant changes in the biochemical parameters related to cardiovascular risk, including serum lipids, over time. There was a significant increase in total, free, and bioavailable testosterone, while SHBG levels significantly decreased; the free androgen index, urine androstanediol glucuronide, androstenedione, and prostate-specific antigen as a marker of androgenization also significantly increased over time. Among the 20 evaluable individuals, 14 (70%) scored ≥4 on the appearance congruence subscale, and 16 (80%) scored ≥4 on the gender identity subscale of the TCS. **Conclusions**: Our results suggest that this conservative treatment paradigm with testosterone is associated with a reduced adverse impact on hematocrit and lipids but is sufficient to achieve the gender congruence and identity acceptance desired by transgender males.

## 1. Introduction

Transgender and gender diverse (TGD) people represent 0.3–0.5% of adults and 1.2–2.7% of children and adolescents, according to survey-based studies in the general population [1]. Health care systems should provide medically necessary gender-affirming health care to TGD people, which includes a variety of medical, psychological, and social interventions that help align a person’s body and lived experience with their gender identity [1]. A key component of these interventions is gender-affirming hormone therapy (GAHT), which is used by approximately 80% of binary transgender people [2]; in transgender males, it consists of the administration of testosterone.

Gender-affirming therapy, including GAHT, improves the quality of life and well-being of TGD people [3,4]. However, GAHT in transgender males could be associated with increased hematocrit [5], erythrocytosis [6], dyslipidemia [7], and subclinical atherosclerosis [8], and although the evidence is more limited, the risk of adverse cardiovascular outcomes is greater in transgender males than in cisgender females [9]. Clinical practice guidelines consistently recommend regular cardiovascular risk assessment and appropriate cardiovascular and cerebrovascular management [1]. Guidelines suggest monitoring potential adverse changes in response to GAHT and monitoring sex hormone levels every 3 months for the first year and once or twice a year afterward [1,10].

Despite the relevance of GAHT with testosterone in transgender males, there is no standardized protocol on how to dose and monitor testosterone therapy, other than dose ranges for testosterone depending on the formulation and maintaining testosterone levels within the normal physiologic range for the affirmed gender [1,10]. Although total testosterone is the recommended biomarker for adjusting hormone therapy, there is no recommendation on which type of assay—immunoassay or mass spectrometry—is needed [11]. The most common method is an immunoassay, but it has poor accuracy when the levels of testosterone are low, and its performance varies across manufacturers [12,13]. Searching for other biomarkers that could guide GAHT in transgender males could help to optimize the management of these therapies. The synthesis of prostate-specific antigen (PSA) in Skene’s gland in women has been described. This synthesis is directly stimulated by testosterone; therefore, elevated levels of PSA in transgender males could serve as biomarkers of androgenization in these individuals [14,15].

The objective of this prospective observational study was to evaluate the hormonal, hematologic, and biochemical outcomes of a conservative protocol for androgen therapy for transgender males that was implemented in our center. We also explored the use of PSA as a biomarker of androgenization.

## 2. Materials and Methods

This was an observational, prospective, and comparative study conducted in the gender identity clinic of a tertiary university hospital. The study was approved by the Research Ethics Committee of Cantabria (Code 2021.206). Each participant provided written informed consent.

### 2.1. Participants

We consecutively recruited transgender males aged ≥18 years who had attended our clinic and were naive to hormonal therapy. There were no exclusion criteria. We also recruited a sample of age-matched cisgender females and cisgender males from hospital medical residents and blood donors at the medical school. Cisgender individuals had to be aged ≥18 years, and in the case of cisgender females, they had to be in the initial phase of the follicular menstrual cycle.

### 2.2. Testosterone Therapy

Testosterone therapy consisted of testosterone cypionate, which is the oil-soluble 17-(beta)-cyclopentylpropionate ester of the androgenic hormone testosterone with a half-life of approximately 8 days. Testosterone cypionate (Desma laboratory, Madrid, Spain) was administered intramuscularly every month at an initial dosage of 25 mg/month, which was increased to 50 mg/month at month 3, 100 mg/month at month 6, and 250 mg/month at month 9. Transgender males were maintained on this latter dose for the rest of the study and as the standard dose for GAHT. The first administration of testosterone was performed within 15 days after the baseline visit, once the laboratory results were available (see below). When hematocrit levels rose, based on clinical judgment, testosterone dose titration was interrupted and maintained stable for three more months while waiting for hematocrit levels to normalize.

### 2.3. Assessments, Sample Collection, and Analyses

Transgender males were seen at baseline and at 3, 6, 12, and 16 months. At baseline, we recorded basic demographic information, medical history, and physical examination findings. Assessments in cisgender females and males were performed only at baseline.

Venous and 24 h urine samples were collected at each study time point and stored at −80 °C until analysis; blood samples for testing were collected three weeks after administering the corresponding testosterone dose, thus corresponding to trough levels. Analyses included a complete blood count, biochemical tests, urine analysis, and hormonal tests.

Basic biochemistry, complete blood count, and coagulation parameters were determined in an automated and standardized manner using Atellica autoanalyzers by Siemens Healthineers^®^ (Forchheim, Germany) and DXH 800 autoanalyzers by Beckman Coulter^®^ (Brea, CA, USA).

Analytical determinations of steroid hormones were performed using a Maglumi 2000 analyzer by Snibe^®^ (Shenzhen, China), except for androstenedione, which was measured by radioimmunoassay using a Beckman Coulter kit (Brea, CA, USA), and androstanediol glucuronide, which was determined by ELISA using a DRG kit (Marburg, Germany).

Prostate-specific antigen (PSA) levels in 24-h urine samples were analyzed using Atellica analyzers. Free testosterone levels were calculated using the Vermeulen formula, bioavailable testosterone was determined using the Morris formula, and the free androgen index was calculated as the ratio of total testosterone to sex hormone-binding globulin (SHBG) [16,17,18].

The laboratory that performed the analyses participated successfully in the external quality assessment program of the Spanish Society of Laboratory Medicine (SEQC) for all the parameters included in the study.

Finally, although not initially foreseen in the protocol, to evaluate transgender congruence and identity acceptance after GAHT, we administered the Transgender Congruence Scale (TCS) after the end of the follow-up. This consisted of a 12-item scale that is intended to measure the degree to which transgender individuals feel genuine, authentic, and comfortable with their gender identity and external appearance [19]. Each item is scored using a 5-point Likert scale of agreement, and the scale measures two factors: appearance congruence (Items 1–9) and gender identity acceptance (Items 10–12). Higher scores indicate higher congruence/acceptance. The scale has not been validated in Spanish, and we used a simple translation of the original scale.

### 2.4. Statistical Analysis

We used a convenience sample of 40 transgender males, 20 cisgender females, and 20 cisgender males.

Descriptive statistics included the mean and standard deviation or the median (P25, P75) for variables that did not follow a normal distribution. Changes in all analytical parameters over time were evaluated using repeated-measures ANOVA. To compare biomarkers of androgenization between transgender males and cisgender females or cisgender males, individuals were age-matched and compared using the Wilcoxon test. To evaluate the association between testosterone forms and potential biomarkers of androgenization (namely, hematocrit, serum PSA, and urine PSA), bootstrap sampling was used to calculate the percentiles. A correlation of 0.40–0.69 was considered moderate, 0.70–0.89 was considered strong, and a correlation ≥0.90 was considered very strong [20]. To show the course in transgender males of the levels of total testosterone, free testosterone, bioavailable testosterone, creatinine, hematocrit, and HDL cholesterol over time with respect to the median levels in cisgender females and cisgender males, we show the 25th, 50th, and 75th percentiles of those biomarkers by means of a boxplot.

To analyze the TCS, we calculated the mean and standard deviation of the two factors of the scale as well as the number of individuals who scored 4 or more points on each subscale.

All analyses were performed with SPSS 25.0. For the changes in laboratory parameters over time, the significance was set at *p* < 0.05. No correction for multiplicity was performed.

## 3. Results

From September 2021 to November 2023, we recruited 40 transgender males with a mean (SD) age of 22.5 (8.4). Two transgender males had comorbidities: one exhibited obesity, hypertension, and diabetes, and the other had hypertension. In addition, 13 individuals had a body mass index (BMI) >25 kg/m^2^, including 6 individuals with overweight and 7 individuals with obesity. All participants received all prescribed doses of testosterone.

The control group consisted of 20 cisgender males, aged 24.5 (11.6), and 20 cisgender females, aged 23.7 (8.2).

### 3.1. Changes in Laboratory Results Among Transgender Males

Among the results of complete blood count parameters, transgender males showed significant increases in erythrocyte, hematocrit, and hemoglobin levels through the 12-month follow-up (Table 1 and Table S1). Pairwise comparisons revealed that only changes in the hematocrit were statistically significant as early as month 9. There were no significant changes in the biochemical parameters related to cardiovascular risk, including serum lipids, over time (Table S2).

No significant changes in the levels of gonadotropins, estradiol, or progesterone were detected (Table S3). There was a significant increase in total, free, and bioavailable testosterone, while SHBG levels significantly decreased; the free androgen index, urine androstanediol glucuronide (3 alpha-diol G), and androstenedione also significantly increased over time (Table 1 and Table S3).

The creatinine level significantly increased over time (Table 1). Serum PSA levels, but not urine PSA levels, increased significantly (Table 1 and Table S3).

No significant changes in the biochemical parameters related to cardiovascular risk, including serum lipids, were observed over time (Table S2).

### 3.2. Comparison of Sex Steroids, Other Potential Biomarkers of Androgenization, and Lipids Between Transgender Males, Cisgender Males, and Cisgender Females

Compared with cisgender males, transgender males showed significant differences in hematocrit levels, PSA levels, creatinine levels, and all sexual steroids at baseline but not in the lipid profile (Table 2). The statistically significant differences were lost at the end of the follow-up (16 months) for total testosterone, free testosterone, and bioavailable testosterone, whereas they were maintained for dehydroepiandrosterone sulfate, free androgen index, hematocrit, PSA, and creatinine. The lack of differences in the lipid profile between transgender males and cisgender males was maintained until the end of the follow-up period.

When compared to cisgender females, at baseline, there were no statistically significant differences in any of the parameters except for high-density lipoprotein (HDL) cholesterol among transgender males (Table 3). At the end of the follow-up period, there were statistically significant differences in all the parameters except for DHEA-S, triglyceride levels, total cholesterol levels, and LDL levels. Differences were observed as early as month 3 for hematocrit, PSA, and the free androgen index and from month 6 onwards for all forms of testosterone.

### 3.3. Correlations Between Biomarkers of Androgenization

At month 9, the correlation between any form of testosterone and serum PSA was statistically significant and of moderate size. There was no correlation between any form of testosterone and urine PSA or hematocrit (Table 4). At month 12, there were no correlations other than among testosterone forms.

### 3.4. Course of Biomarkers of Androgenization in Transgender Males with Respect to Median Values of Cisgender Males and Cisgender Females

**Figure 1:**
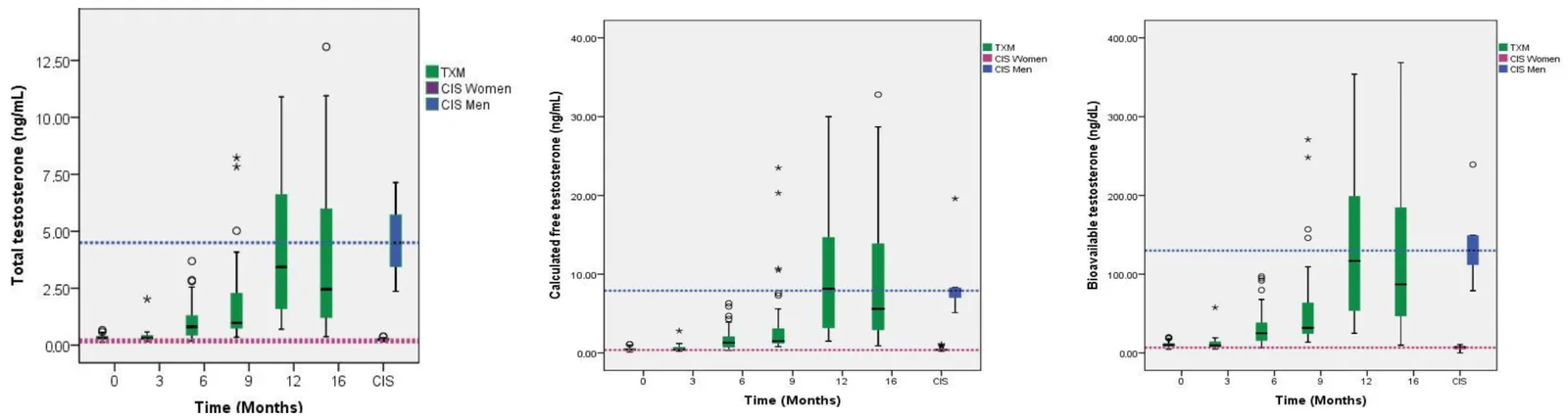
Course of biomarkers of androgenization in transgender males with respect to median values of cisgender males. Asterisks (*) and circles (○) indicate extreme and moderate outliers, respectively. Creatinine levels in transgender males were generally above the median for cisgender females and below the median for cisgender males at all time points (Figure 2). Once the maximum testosterone dose was reached (i.e., at month 12), more than 75% of the transgender males had a hematocrit level above the median for cisgender males (Figure 2). HDL-C levels in transgender males were generally below the median in cisgender females at all time points, and in more than 50% of them, they were also below the median in cisgender males at baseline and at all follow-up time points.

Serum testosterone levels in transgender males were generally below the median for cisgender males for all forms of testosterone (Figure 1). However, for free and bioavailable testosterone, once the maximum testosterone dose was reached (i.e., 250 mg at month 12), a fraction of transgender males between 25% and 50% were above the median levels for cisgender males (Figure 1).

**Figure 2:**
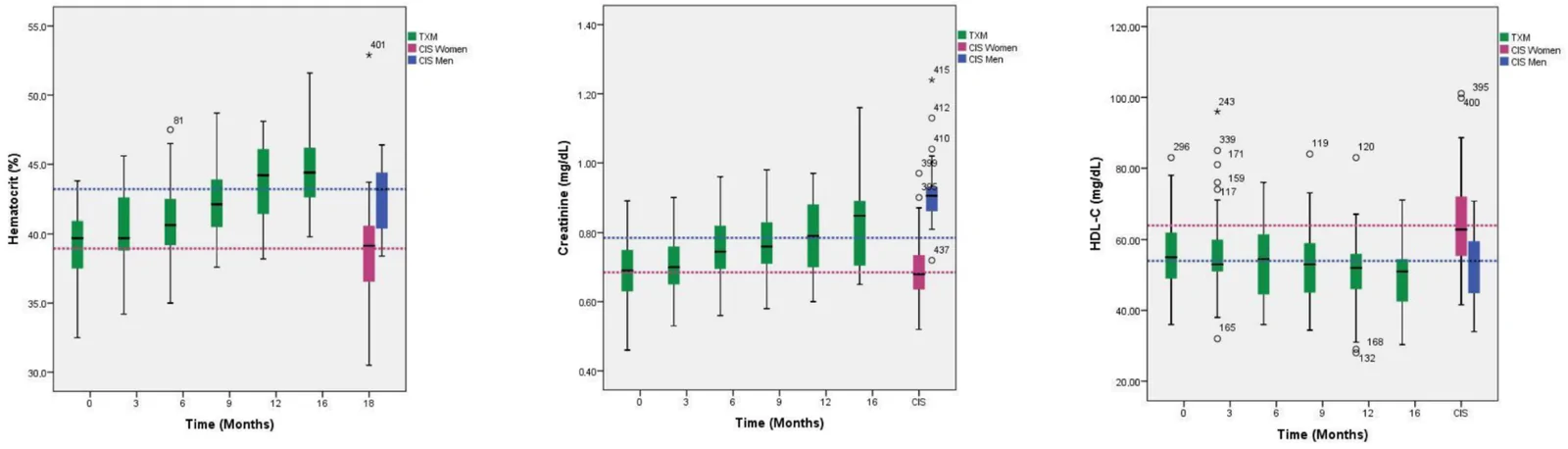
Course of biomarkers of androgenization in transgender males with respect to median values of cisgender females. Asterisks (*) and circles (○) indicate extreme and moderate outliers, respectively.

### 3.5. Other Safety Evaluations

The following adverse events were reported during the follow-up: anxiety (*n* = 3), hypertension (*n* = 2), subclinical hypothyroidism (*n* = 2), osteopenia (*n* = 1), acne (*n* = 1), and anorexia (*n* = 1).

Diastolic blood pressure increased over time from a mean (SD) of 75 (14) mmHg at baseline to 84 (14) mmHg at month 12. Systolic blood pressure increased over time from a mean of 123 (20) mmHg at baseline to 131 (16) mmHg at month 12. BMI also increased from a mean of 24.5 (7.7) kg/m^2^ at baseline to 26.7 (7.5) kg/m^2^ at month 12. None of these changes over time were statistically significant (Table 5).

### 3.6. Post Hoc Evaluation of Transgender Identity Congruence and Identity Acceptance

Twenty participants completed the scale. All individuals answering the questionnaire were under the androgenization protocol with a minimum time on the maintenance dose (i.e., 250 mg) of 4 months. The mean (SD) score on the two subscales of the TCS was 4.08 (0.11) on the appearance congruence subscale and 4.31 (0.29) on the gender identity subscale. The number of individuals who scored 4 or more points was 14 (70%) on the appearance congruence subscale and 16 (80%) on the gender identity subscale.

### 3.7. Menstrual Suppression

Menstrual suppression was achieved in 35 transgender males: in 25 individuals at a dose of 250 mg/month, in 8 individuals at 100 mg/month, and in 2 individuals at 50 mg/month. In two transgender males, menstrual suppression was not achieved during the study period; in one individual, amenorrhea was present at the time of study initiation; and in two individuals, we could not retrieve information on menstrual suppression.

## 4. Discussion

The conservative androgenization protocol used in our study was associated with a significant increase in hematocrit and no significant changes in metabolic parameters related to cardiovascular risk, including the lipid profile. On the basis of our post hoc analysis, our results suggest that a substantial proportion of transgender males were comfortable with their appearance and gender identity.

An increase in hematocrit is considered the highest risk factor for adverse medical outcomes associated with testosterone therapy in transgender males [10]. In our cohort, we found a significant increase in hematocrit, from a mean of 39.4% ± 2.4 to 43.8% ± 2.6 at month 12. Several systematic reviews have consistently reported an increase in hematocrit levels in transgender males under testosterone treatment [5,6,21], although only one of them conducted a meta-analysis [21]. Tienforti et al. pooled data from 6 studies and reported a mean difference with respect to baseline in the hematocrit of 4.49% (95% confidence interval, 3.52 to 5.26) [21]. Our findings, a difference of 4.4%, are consistent with those of this meta-analysis. However, it is important to note that among the 6 studies included in that meta-analysis, three evaluated testosterone undecanoate, one evaluated testosterone enanthate depot, and one evaluated testosterone gel, which are long-acting testosterone formulations or transdermal gels that could be associated with a lower impact on the hematocrit level. In their systematic review, Okano et al. reported that in studies with testosterone undecanoate, the hematocrit level increased to 5%, whereas in those studies using intermediate injectable testosterone esters, it increased to 6.9% [5]. More specifically, the change from baseline in the hematocrit after one year of treatment with intermediate injectable testosterone esters was 4% in a study in Italy with testosterone enanthate 250 mg i.m. every 28 days [22], 4.5% with testosterone enanthate every 3–4 weeks [23], 5% with Sustanon^®^ (a mixture of different testosterone esters with varying release speeds) 250 mg every 2 weeks [24], 5.1% with Sustanon^®^ at increasing doses up to 125 mg every 2 weeks [25], 6.1% with injectable testosterone mostly every two weeks (mean daily dose 11.4 mg) [26], and 6.9% with several formulations of testosterone [27]. Overall, although under our androgenization protocol, the hematocrit increased significantly and was greater than the level of cisgender males in 75% of the transgender males, the increase appears lower than that reported in most studies using intermediate injectable testosterone esters. The clinical relevance of this increase in hematocrit levels in transgender males is unknown. In cisgender males starting testosterone therapy, an increase in hematocrit has been associated with an increased risk of major adverse cardiovascular events [28]. However, although the evidence is limited, testosterone in transgender males does not appear to be associated with an increased risk of venous thromboembolism [29]. A systematic review comparing transgender people with cisgender people revealed that compared with cisgender females, transgender males had increased risks of stroke (relative risk [RR] 1.3, 95% CI, 1.0–1.6) and venous thromboembolism (RR 1.4, 95% CI, 1.0–2.0) but not of myocardial infarction (RR 1.7, 95% CI, 0.8–3.6) [9]. Overall, although clinical practice guidelines contain only recommendations for monitoring hematocrit levels but do not provide guidance on how to manage hematocrit increase [1,10], the results from the use of testosterone in cisgender males and the limited data on the increased risk of cardiovascular events in transgender males support limiting the impact of testosterone therapy on hematocrit as much as possible.

We did not find significant changes in metabolic parameters related to cardiovascular risk, including the lipid profile. The most consistent effect of testosterone therapy on these parameters is a reduction in high-density lipoprotein (HDL) cholesterol concentration, which is estimated to range from 4 to 13 mg/dL for 1 year [30]. A systematic review of 11 studies revealed that most studies did not report changes in cholesterol or triglyceride levels with hormone therapy, but 7 of the 11 studies reported a decrease in HDL cholesterol levels [7]. In our series, the difference after 1 year with respect to baseline in HDL cholesterol levels was a reduction of 5 mg/dL, that is, within the lower limit reported in the literature. We did not find any significant change in the glycemic parameters or in insulin sensitivity as measured with the homeostatic model assessment-insulin resistance, which remained almost unchanged throughout the study. These latter results are consistent with previous findings [31,32]. Although changes in blood pressure were not statistically significant, there was a trend toward increased values. According to a systematic review, studies evaluating changes in blood pressure in transgender males provide heterogeneous and inconsistent results, with some studies reporting a decrease in diastolic and systolic blood pressure and others reporting an increase in diastolic (ranging from 1.5 to 3.7 mmHg) and systolic (ranging from 1.5 to 13.3 mmHg) blood pressure [33]. Regardless of these inconsistent results, regular assessment of metabolic risk factors, including blood pressure, is recommended to monitor cardiovascular risk.

The trajectory of total, free, and bioavailable testosterone levels, which are directly related to the administration of testosterone, reflects that transgender males differentiated from cisgender females as early as 6 months. However, significant differences in these parameters compared with those of cisgender males were maintained until month 12, and at month 16, the differences were statistically significant only for bioavailable testosterone; in fact, a relevant proportion (25–50%) of transgender males had bioavailable and free testosterone levels above the median levels of cisgender males. The objective of GAHT is to achieve changes in transgender males that are consistent with their embodiment goals and/or gender identity [1]. Unfortunately, in our study, we did not record changes in phenotype or gender congruence, and identity acceptance was evaluated post hoc in only half of the individuals. Despite these limitations, the available results suggest that a substantial proportion (70–80%) of transgender males were comfortable with their appearance and gender identity.

The role of serum PSA as a biomarker of androgenization according to our results is unclear. While we found a moderate and statistically significant correlation between serum PSA concentration and total testosterone concentration at month 9, this correlation was lost at month 12. This loss of correlation may be due to saturation of the tissue’s PSA synthetic capacity, although this is only speculative. In our study, testosterone levels increased substantially between 9 and 12 months, whereas the corresponding increase in PSA was not observed to the same extent. PSA synthesis occurs following the direct action of testosterone on target cells; therefore, it represents a direct marker and may reflect the effects of testosterone more clearly than other markers, such as hematocrit or lipid profiles [14,34]. This loss of correlation could suggest that the doses reached at 12 months exceeded the androgenization capacity of certain tissues. It would be of interest to assess the evolution of this parameter in conjunction with phenotypic changes within a dosing range of 100–250 mg/month.

In addition to the lack of information recorded on the changes in phenotype and the abovementioned limitations on the post hoc evaluation of gender congruence and identity acceptance using an evaluation tool that has not been validated in our setting, our study has other limitations. We did not record information on other patient-reported outcomes, such as quality of life. Information on adverse events was based on spontaneous reports by the participants, which could lead to underreporting of adverse events. The sample size of transgender males was relatively small and thus precluded the detection of small but clinically relevant differences (e.g., changes in blood pressure). We performed multiple comparisons without controlling for multiplicity. Although some authors advocate not using multiplicity correction [35,36], we believe that owing to the risk of inflating type I error, our results should be considered exploratory. Our androgenization protocol was performed at a single center using testosterone cypionate. Therefore, our results cannot be generalized to other centers or testosterone intramuscular formulations. Finally, the lack of a control group with other testosterone regimens and a follow-up limited to 16 months are also limitations of our study design.

Despite these limitations, our results suggest that this conservative treatment paradigm with testosterone is associated with a reduced adverse impact on hematocrit and no relevant impact on lipids but is sufficient to achieve the gender congruence and identity acceptance desired by transgender males. Our evolving laboratory data on several safety parameters and biomarkers of androgenization could help other clinicians implement our conservative protocol for androgenization. These results require confirmation in larger, multicenter studies properly powered to detect clinically relevant differences in safety parameters.

## Figures and Tables

**Table 1:** Summary of significant changes in laboratory parameters.

Parameter	Baseline	Month 3	Month 6	Month 9	Month 12	*p* Value *
Complete blood count and biochemical parameters						
Hematocrit (%)	39.4 ± 2.4 39.7 (3.8)	39.7 ± 2.7 39.1 (4.5)	40.6 ± 2.9 41.1 (4.0)	42.3 ± 2.5 42.1 (3.0)	43.8 ± 2.6 44.3 (4.2)	*p* < 0.001 0–9: *p* = 0.006 0–12: *p* < 0.001
Serum prostate-specific antigen (ng/mL)	0.023 ± 0.015 0.02 (0.02)	0.032 ± 0.02 0.025 (0.02)	0.040 ± 0.03 0.030 (0.04)	0.041 ± 0.04 0.035 (0.03)	0.048 ± 0.03 0.040 (0.04)	* *p* < 0.001 0–12: * *p* < 0.001
Creatinine (mg/dL)	0.70 ± 0.09 0.69 (0.15)	0.70 ± 0.09 0.70 (0.15)	0.74 ± 0.09 0.73 (0.15)	0.77 ± 0.10 0.75 (0.19)	0.79 ± 0.11 0.79 (0.22)	*p* < 0.001 0–12: *p* = 0.027
Sexual steroids						
Free testosterone (pg/mL)	3.0 ± 1.2 2.8 (1.4)	3.4 ± 1.2 3.1 (1.5)	9.1 ± 6.9 6.9 (4.3)	10.8 ± 13.9 6.6 (4.8)	26.2 ± 19.9 20.8 (30)	* *p* < 0.001 * 0–12: *p* < 0.001
Total testosterone (ng/mL)	0.34 ± 0.12 0.33 (0.12)	0.39 ± 0.32 0.33 (0.19)	1.12 ± 0.86 0.84 (1.03)	1.85 ± 1.98 0.97 (1.76)	4.35 ± 3.07 3.17 (5.11)	* *p* < 0.001 * 0–12: *p* < 0.001
Free testosterone (Vermeulen) (ng/dL)	0.49 ± 0.24 0.45 (0.3)	0.56 ± 0.49 0.40 (0.48)	1.94 ± 1.59 1.40 (2.0)	3.82 ± 5.30 1.50 (3.55)	10.4 ± 8.30 7.90 (12.6)	* *p* < 0.001 0–12: *p* < 0.001
Bioavailable testosterone (Morris) (ng/dL)	10.8 ± 3.9 10.2 (4.7)	12.3 ± 9.3 9.4 (8.4)	35.6 ± 25.9 26.5 (32.1)	58.6 ± 63.0 28.8 (54.1)	140.5 ± 97.3 108.9 (157.9)	*p* < 0.001 0–12: *p* < 0.001
Sex hormone-binding globulin (nmol/L)	56.9 ± 41.0 47.5 (46)	53.3 ± 26.6 50.0 (50)	39.6 ± 19.3 34.0 (23)	35.3 ± 17.2 29.5 (30)	27.4 ± 11.4 25.5 (16)	* *p* < 0.001 0–9: *p* = 0.025 0–12: *p* < 0.001
Free androgen index (%)	2.8 ± 1.9 2.7 (1.8)	3.3 ± 2.9 2.2 (2.9)	11.9 ± 10.9 8.3 (10.5)	22.4 ± 29.8 10.2 (16.3)	64.2 ± 54.6 54.0 (72.0)	* *p* < 0.001 0–12: *p* < 0.001
Urine androstanediol glucuronide (ng/mL)	231 ± 173 158 (275)	200 ± 182 148 (148)	271 ± 171 201 (256)	393 ± 384 245 (412)	490 ± 516 347 (300)	*p* = 0.032 0–9: *p* = 0.028 0–12: *p* = 0.018
Androstenedione (ng/mL)	2.93 ± 1.07 2.80 (1.3)	2.96 ± 0.90 2.90 (1.3)	3.08 ± 1.00 3.05 (1.8)	3.47 ± 1.05 3.45 (2.7)	4.03 ± 1.36 4.10 (2.7)	* *p* < 0.001 0–9: *p* = 0.028 0–12: *p* = 0.015

The results are shown as the means ± standard deviations and median (IQR). * *p* values correspond to the following: the upper *p* value is the result of the ANOVA test, and the other *p* values are those of the post hoc Scheffe test, but we are only presenting those that were statistically significant. Missing data for serum parameters and hematocrit were present in 2 individuals at months 3, 6, and 12 and in 3 individuals at month 9. Missing data for urine analyses were present in 16 individuals at baseline, 19 individuals at months 3 and 9, 27 individuals at month 6, and 25 individuals at month 12.

**Table 2:** Comparison of key parameters of androgenization and lipids between transgender males and cisgender males.

Parameter	Baseline	Month 3	Month 6	Month 9	Month 12	Month 16
Hematocrit	*p* < 0.001	*p* < 0.001	*p* = 0.003	*p* = 0.315	*p* = 0.426	*p* = 0.018
PSA	*p* < 0.001	*p* < 0.001	*p* < 0.001	*p* < 0.001	*p* < 0.001	*p* < 0.001
Creatinine	*p* < 0.001	*p* < 0.001	*p* < 0.001	*p* < 0.001	*p* < 0.001	*p* = 0.023
Total testosterone	*p* < 0.001	*p* < 0.001	*p* < 0.001	*p* < 0.001	*p* = 0.039	*p* = 0.161
Free testosterone	*p* < 0.001	*p* < 0.001	*p* = 0.001	*p* = 0.007	*p* = 0.057	*p* = 0.058
Bioavailable testosterone	*p* < 0.001	*p* < 0.001	*p* < 0.001	*p* = 0.010	*p* = 0.131	*p* = 0.026
DHEA-S	*p* < 0.001	*p* = 0.001	*p* = 0.009	*p* = 0.007	*p* = 0.005	*p* = 0.039
Free androgen index	*p* < 0.001	*p* < 0.001	*p* < 0.001	*p* = 0.376	*p* = 0.002	*p* = 0.015
Triglycerides	*p* = 0.027	*p* = 0.050	*p* = 0.041	*p* = 0.107	*p* = 0.306	*p* = 0.232
Total cholesterol	*p* = 0.997	*p* = 0.287	*p* = 0.405	*p* = 0.189	*p* = 0.141	*p* = 0.843
HDL-C	*p* = 0.282	*p* = 0.298	*p* = 0.669	*p* = 0.639	*p* = 0.929	*p* = 0.153
LDL-C	*p* = 0.229	*p* = 0.184	*p* = 0.577	*p* = 0.690	*p* = 0.820	*p* = 0.173

DHEA-S, dehydroepiandrosterone sulfate; HDL-C, high-density lipoprotein cholesterol; LDL-C, low-density lipoprotein cholesterol; PSA, prostate-specific antigen.

**Table 3:** Comparison of key parameters of androgenization and lipids between transgender males and cisgender females.

Parameter	Baseline	Month 3	Month 6	Month 9	Month 12	Month 16
Hematocrit	*p* = 0.688	*p* = 0.014	*p* = 0.029	*p* < 0.001	*p* < 0.001	*p* < 0.001
PSA	*p* = 0.068	*p* = 0.026	*p* = 0.019	*p* = 0.002	*p* = 0.004	*p* = 0.011
Creatinine	*p* = 0.712	*p* = 0.972	*p* = 0.055	*p* = 0.004	*p* = 0.002	*p* < 0.001
Total testosterone	*p* = 0.856	*p* = 0.993	*p* = 0.002	*p* = 0.002	*p* < 0.001	*p* < 0.001
Free testosterone	*p* = 0.916	*p* = 0.511	*p* < 0.001	*p* = 0.001	*p* < 0.001	*p* < 0.001
Bioavailable testosterone	*p* = 0.308	*p* = 0.061	*p* = 0.001	*p* = 0.003	*p* < 0.001	*p* = 0.004
DHEA-S	*p* = 0.068	*p* = 0.385	*p* = 0.770	*p* = 0.953	*p* = 0.955	*p* = 0.111
Free androgen index	*p* = 0.107	*p* = 0.002	*p* < 0.001	*p* < 0.001	*p* < 0.001	*p* < 0.001
Triglycerides	*p* = 0.061	*p* = 0.052	*p* = 0.039	*p* = 0.185	*p* = 0.480	*p* = 0.231
Total cholesterol	*p* = 0.086	*p* = 0.267	*p* = 0.311	*p* = 0.862	*p* = 0.417	*p* = 0.660
HDL-C	*p* < 0.001	*p* = 0.018	*p* = 0.001	*p* < 0.001	*p* < 0.001	*p* = 0.003
LDL-C	*p* = 0.763	*p* = 0.495	*p* = 0.222	*p* = 0.073	*p* = 0.185	*p* = 0.219

DHEA-S, dehydroepiandrosterone sulfate; HDL-C, high-density lipoprotein cholesterol; LDL-C, low-density lipoprotein cholesterol; PSA, prostate-specific antigen.

**Table 4:** Correlations between biomarkers of androgenization at 9 months and 12 months.

Parameter	Total Testosterone	Free Testosterone	PSA-Serum	PSA-Urine	Hematocrit
	Month 9				
Total testosterone		*p* < 0.001	*p* = 0.008	*p* = 0.993	*p* = 0.881
		rho = 0.901	rho = 0.417	rho = 0.002	rho = 0.25
		*n* = 40	*n* = 39	*n* = 22	*n* = 38
Free testosterone	*p* < 0.001		*p* = 0.004	*p* = 0.994	*p* = 0.718
	rho = 0.901		rho = 0.449	rho = 0.002	rho = 0.060
	*n* = 40		*n* = 39	*n* = 22	*n* = 38
Bioavailable testosterone	*p* < 0.001	*p* < 0.001	*p* = 0.003	*p* = 0.984	*p* = 0.892
	rho = 0.973	rho = 0.955	rho = 0.463	rho = 0.05	rho = 0.023
	*n* = 40	*n* = 40	*n* = 35	*n* = 22	*n* = 38
	Month 12				
Total testosterone		*p* < 0.001	*p* = 0.909	*p* = 0.485	*p* = 0.415
		rho = 0.977	rho = 0.020	rho = 0.188	rho = 0.1422
		*n* = 38	*n* = 35	*n* = 16	*n* = 40
Free testosterone	*p* < 0.001		*p* = 0.596	*p* = 0.474	*p* = 0.316
	rho = 0.977		rho = 0.093	rho = 0.193	rho = 0.175
	*n* = 38		*n* = 35	*n* = 16	*n* = 40
Bioavailable testosterone	*p* < 0.001	*p* < 0.001	*p* = 0.785	*p* = 0.594	*p* = 0.446
	rho = 0.991	rho = 0.993	rho = 0.049	rho = 0.150	rho = 0.135
	*n* = 38	*n* = 38	*n* = 35	*n* = 16	*n* = 40

**Table 5:** Changes in blood pressure and body mass index over time.

	Baseline	Month 3	Month 6	Month 9	Month 12
Diastolic blood pressure (mmHg)	75 ± 14 75 (26)	78 ± 15 78 (17)	73 ± 14 77 (19)	80 ± 13 78 (20)	84 ± 14 83 (14)
Systolic blood pressure (mmHg)	123 ± 20 120 (33)	126 ± 22 124 (34)	129 ± 16 125 (17)	133 ± 15 128 (23)	131 ± 16 125 (25)
Body mass index (kg/m^2^)	24.5 ± 7.7 22.9 (9.3)	24.9 ± 6.9 24.1 (7.9)	24.9 ± 5.6 22.8 (9.0)	27.8 ± 6.9 23.8 (10.9)	26.7 ± 7.5 24.1 (9.8)

All figures are mean ± standard deviation and median (interquartile range). None of the changes over time were statistically significant (Kruskal–Wallis).

## Data Availability

The original contributions presented in this study are included in the article. Further inquiries can be directed to the corresponding author.

## Supplementary Materials

The following supporting information can be downloaded at https://www.mdpi.com/article/10.3390/jcm15166498/s1. Table S1: Changes in biochemical parameters other than those related to cardiovascular risk, complete blood count, and coagulation parameters over time; Table S2: Changes in biochemical parameters related to cardiovascular risk over time; Table S3: Changes over time in gonadotropins, sex steroids, and prostate-specific antigens.
