# Supplementary material for: Course of Testosterone Levels and Potential Biochemical Biomarkers of Androgenization with a Conservative Protocol of Androgen Hormone Therapy in Transgender Males
Source: J Clin Med. 2026 Aug 21;15(16):6498. doi: 10.3390/jcm15166498 (PMC13514267; doi:10.3390/jcm15166498)
Supplement: Supplementary file 1 [file jcm-15-06498-s001.zip › jcm-4425019-supplementary.pdf]

Supplementary Materials

Table S1. Changes in biochemical parameters other than those related to cardiovascular risk, complete blood count, and coagulation parameters over time.

Parameter	Baseline	Month 3	Month 6	Month 9	Month 12	p value*
**Glucose (mg/dL)**	88 ± 8<br>88 (9)	86 ± 14<br>84 (7)	87 ± 7<br>87 (10)	86 ± 9<br>86 (8)	87 ± 9<br>87 (11)	p=0.971
**Creatinine (mg/dL)**	0.70 ± 0.09<br>0.69 (0.15)	0.70 ± 0.09<br>0.70 (0.15)	0.74 ± 0.09<br>0.73 (0.15)	0.77 ± 0.10<br>0.75 (0.19)	0.79 ± 0.11<br>0.79 (0.22)	p<0.001<br>0-12: p=0.027
**Urea (mg/dL)**	27.1 ± 5.8<br>26 (8)	26.93 ± 5.74<br>28 (6)	29.2 ± 7.8<br>28 (8)	28.8 ± 6.8<br>27 (11)	27.8 ± 7.1<br>27 (12)	p=0.546
**Calcium (mg/dL)**	9.5 ± 0.5<br>9.5 (0.6)	9.6 ± 0.5<br>9.6 (0.8)	9.6 ± 0.5<br>9.4 (0.8)	9.7 ± 0.4<br>9.7 (0.5)	9.6 ± 0.4<br>9.6 (0.55)	p=0.562
**Albumin (mg/dL)**	4.6 ± 0.3<br>4.5 (0.3)	4.6 ± 0.3<br>4.6 (0.4)	4.6 ± 0.3<br>4.6 (0.3)	4.6 ± 0.2<br>4.6 (0.4)	4.6 ± 0.3<br>4.6 (0.4)	p=0.270
**ALT (IU/L)**	18.7 ± 11.7<br>14 (10)	16.6 ± 8.1<br>15 (9)	20.0 ± 14.9<br>15 (11)	23.0 ± 18.7<br>18 (12)	21.0 ± 10.8<br>17 (13)	p=0.211
**AST (IU/L)**	21.0 ± 5.5<br>19 (7)	19.3 ± 3.9<br>18 (5)	21.1 ± 6.1<br>19 (8)	22.0 ± 12.7<br>20 (6)	23.0 ± 9.8<br>21 (8)	p=0.276
**GGT (IU/L)**	16.4 ± 7.9<br>14 (8)	16.4 ± 7.2<br>15 (8)	16.9 ± 6.1<br>15 (10)	21.8 ± 18.4<br>18 (12)	18.3 ± 6.5<br>18 (9)	p=0.102
**ALP (IU/L)**	70.7 ± 22.4<br>65 (19)	68.8 ± 17.1<br>66 (16)	70.2 ± 20.2<br>67 (22)	76.8 ± 28.7<br>71 (27)	74.7 ± 24.5<br>72 (25)	p=0.605
**Total bilirubin (mg/dL)**	0.5 ± 0.2<br>0.4 (0.3)	0.51 ± 0.23<br>0.5 (0.4)	0.50 ± 0.17<br>0.5 (0.2)	0.53 ± 0.2<br>0.5 (0.2)	0.59 ± 0.23<br>0.5 (0.4)	p=0.500
**TSH (mIU/L)**	2.41 ± 1.72<br>1.91 (1.81)	2.12 ± 1.32<br>1.89 (1.64)	2.48 ± 1.85<br>2.11 (1.70)	2.64 ± 1.75<br>2.41 (1.90)	2.45 ± 1.33<br>2.3 (1.24)	p=0.776
**Prolactin (ng/mL)**	13.9 ± 7.0<br>10.9 (8.6)	13.8 ± 9.3<br>11.8 (6.4)	11.1 ± 5.0<br>9.9 (6.7)	10.8 ± 4.1<br>9.9 (5.6)	10.7 ± 3.7<br>10.0 (6.6)	p=0.026

**Cortisol (μg/dL)**	19.5 ± 6.6<br>19.0 (8.1)	19.8 ± 6.6<br>19.9 (10.4)	18.8 ± 6.1<br>18.8 (7.0)	20.0 ± 5.1<br>20.1 (4.9)	19.6 ± 5.9<br>19.5 (7.2)	p=0.982
**Erythrocytes (x 10^6)**	4.48 ± 0.31<br>4.50 (0.27)	4.51 ± 0.31<br>4.46 (0.39)	4.63 ± 0.32<br>4.62 (0.46)	4.76 ± 0.32<br>4.76 (0.58)	4.97 ± 0.32<br>5.04 (0.44)	p<0.001<br>0-12: p<0.001
**Hematocrit (%)**	39.4 ± 2.4<br>39.7 (3.8)	39.7 ± 2.7<br>39.1 (4.5)	40.6 ± 2.9<br>41.1 (4.0)	42.3 ± 2.5<br>42.1 (3.0)	43.8 ± 2.6<br>44.3 (4.2)	p<0.001<br>0-9: p=0.006<br>0-12: p<0.001
**Hemoglobin (g/dL)**	13.3 ± 1.0<br>13.5 (1.5)	13.4 ± 1.1<br>13.3 (1.8)	13.5 ± 1.2<br>13.7 (1.7)	14.1 ± 0.9<br>14.1 (1.5)	14.6 ± 0.99<br>14.6 (1.4)	p<0.001<br>0-12: p=0.003
**D-dimer (ng/mL)**	418 ± 86<br>263 (102)	280 ± 169<br>224 (154)	246 ± 135<br>227 (135)	284 ± 201<br>229 (120)	224 ± 156<br>181 (98)	p=0.119
**Platelets (10^9/L)**	243 ± 62<br>244 (94)	249 ± 60<br>246 (87)	250 ± 61<br>236 (98)	262 ± 69<br>251 (101)	258 ± 61<br>235 (98)	p=0.853
**Activated partial thromboplastin time (s)**	29.4 ± 2.2<br>29.4 (2.8)	29.7 ± 2.2<br>29.6 (3.5)	31.4 ± 8.4<br>29.7 (3.0)	29.8 ± 2.8<br>29.4 (3.5)	29.9 ± 2.2<br>29.7 (3.1)	p=0.326
**Thrombin time (s)**	12.3 ± 0.9<br>12.2 (1.3)	12.2 ± 1.0<br>12.0 (1.2)	12.2 ± 0.9<br>12.2 (1.4)	12.2 ± 0.9<br>12.3 (1.2)	12.0 ± 0.9<br>11.9 (1.3)	p=0.474
**Fibrinogen (mg/dL)**	413 ± 80<br>389 (102)	420 ± 86<br>418 (154)	420 ± 83<br>390 (135)	424 ± 89<br>406 (120)	388 ± 72<br>381 (98)	p=0.341

ALP, alkaline phosphatase; ALT, alanine aminotransferase; AST, aspartate aminotransferase; GGT, gamma-glutamyl transferase.

* p values correspond to the following: the upper p value is the result of the ANOVA test, and the other p values are those of the post hoc Scheffe test, but we are only presenting those that were statistically significant.

Table S2. Changes in biochemical parameters related to cardiovascular risk over time.

Parameter	Baseline	Month 3	Month 6	Month 9	Month 12	p value*
Insulin (mIU/mL)	11.8 ± 9.5<br>10.0 (5.7)	12.4 ± 8.5<br>10.7 (8.3)	10.9 ± 6.6<br>9.5 (7.0)	11.3 ± 7.0<br>10.0 (6.9)	12.0 ± 8.3<br>10.9 (5.8)	p=0.986
HbA1c (%)	5.2 ± 0.3<br>5.1 (0.30)	5.2 ± 0.3<br>5.3 (0.35)	5.2 ± 0.3<br>5.2 (0.35)	5.2 ± 0.3<br>5.3 (0.52)	5.2 ± 0.4<br>5.2 (0.45)	p=0.976
HOMA-IR	2.61 ± 2.28<br>2.26 (1.3)	2.63 ± 1.72<br>2.30 (2.2)	2.34 ± 1.31<br>2.06 (1.6)	2.46 ± 1.67<br>2.12 (1.5)	2.60 ± 1.76<br>2.29 (1.7)	p=0.983
Total cholesterol (mg/dL)	166 ± 31<br>155 (45)	164 ± 29<br>157 (46)	165 ± 126<br>165 (39)	169 ± 34<br>164 (39)	162 ± 38<br>160 (44)	p=0.954
Triglycerides (mg/dL)	75 ± 32<br>72 (31)	79 ± 38<br>64 (54)	70 ± 19<br>70 (29)	82 ± 31<br>79 (40)	83 ± 33<br>78 (38)	p=0.496
HDL-C (mg/dL)	55 ± 10<br>56 (14)	54 ± 10<br>53 (9)	55 ± 11<br>56 (20)	52 ± 12<br>51 (14)	50 ± 10<br>51 (14)	p=0.118
LDL-C (mg/dL)	96 ± 27<br>93 (36)	94 ± 25<br>89 (24)	95 ± 29<br>90 (28)	101 ± 31<br>100 (42)	96 ± 24<br>95 (22)	p=0.642

HOMA-IR, Homeostatic Model Assessment of Insulin Resistance; HDL-C, High-density lipoprotein cholesterol; LDL-C, Low-density lipoprotein cholesterol.

* p values correspond to the following: the upper p value is the result of the ANOVA test, and the other p values are those of the post hoc Scheffe test, but we are only presenting those that were statistically significant.

Table S3. Changes over time in gonadotropins, sex steroids and prostate-specific antigens.

Parameter	**Baseline**	**Month 3**	**Month 6**	**Month 9**	**Month 12**	**p value**
**LH (mIU/mL)**	7.3 ± 7.5<br>5.2 (6.8)	7.5 ± 6.1<br>6.9 (5.8)	7.7 ± 7.4<br>6.5 (6.8)	8.5 ± 7.6<br>6.9 (6.8)	5.5 ± 5.8<br>4.4 (5.0)	p=0.724
**FSH (mIU/mL)**	6.0 ± 4.6<br>5.3 (4.2)	7.3 ± 9.1<br>5.6 (2.3)	8.3 ± 13.0<br>5.9 (3.2)	8.5 ± 12.4<br>6.3 (3.6)	7.5 ± 11.1<br>5.5 (3.4)	p=0.915
**Estradiol (pg/mL)**	92.5 ± 76.5<br>72.0 (66.7)	109.6 ± 103.3<br>70.7 (116.6)	83.7 ± 68.4<br>62.7 (80.9)	90.5 ± 80.0<br>65.4 (77.4)	63.9 ± 51.9<br>54.8 (26.6)	p=0.272
**Progesterone (ng/mL)**	2.5 ± 4.2<br>0.9 (1.7)	2.5 ± 5.5<br>0.9 (1.8)	1.7 ± 2.4<br>1.0 (1.4)	2.1 ± 2.6<br>1.1 (0.5)	1.1 ± 1.1<br>0.88 (0.5)	p=0.399
**17-OHP (ng/mL)**	3.4 ± 9.2<br>1.3 (1.6)	1.8 ± 1.1<br>1.3 (1.2)	1.6 ± 1.0<br>1.3 (1.0)	1.8 ± 1.0<br>1.7 (1.0)	1.4 ± 0.8<br>1.2 (0.9)	p=0.380
**Total testosterone (ng/mL)**	0.34 ± 0.12<br>0.33 (0.12)	0.39 ± 0.32<br>0.33 (0.19)	1.12 ± 0.86<br>0.84 (1.03)	1.85 ± 1.98<br>0.97 (1.76)	4.35 ± 3.07<br>3.17 (5.11)	*p<0.001<br>*0-12: p<0.001
**Free testosterone (pg/mL)**	3.0 ± 1.2<br>2.8 (1.4)	3.4 ± 1.2<br>3.1 (1.5)	9.1 ± 6.9<br>6.9 (4.3)	10.8 ± 13.9<br>6.6 (4.8)	26.2 ± 19.9<br>20.8 (30)	*p<0.001<br>*0-12: p<0.001
**Free testosterone Vermeulen)**	0.49 ± 0.24<br>0.45 (0.3)	0.56 ± 0.49<br>0.40 (0.48)	1.94 ± 1.59<br>1.40 (2.0)	3.82 ± 5.30<br>1.50 (3.55)	10.4 ± 8.30<br>7.90 (12.6)	*p<0.001<br>0-12: p<0.001
**Bioavailable testosterone (Morris)**	10.8 ± 3.9<br>10.2 (4.7)	12.3 ± 9.3<br>9.4 (8.4)	35.6 ± 25.9<br>26.5 (32.1)	58.6 ± 63.0<br>28.8 (54.1)	140.5 ± 97.3<br>108.9 (157.9)	p<0.001<br>0-12: p<0.001
**SHBG (nmol/L)**	56.9 ± 41.0<br>47.5 (46)	53.3 ± 26.6<br>50.0 (50)	39.6 ± 19.3<br>34.0 (23)	35.3 ± 17.2<br>29.5 (30)	27.4 ± 11.4<br>25.5 (16)	*p<0.001<br>0-9: p=0.025<br>0-12: p<0.001
**Free androgen index (%)**	2.8 ± 1.9<br>2.7 (1.8)	3.3 ± 2.9<br>2.2 (2.9)	11.9 ± 10.9<br>8.3 (10.5)	22.4 ± 29.8<br>10.2 (16.3)	64.2 ± 54.6<br>54.0 (72.0)	*p<0.001<br>0-12: p<0.001
**DHEA-S (μg/dL)**	196 ± 85<br>191 (137)	212 ± 93<br>196 (144)	230 ± 91<br>226 (148)	228 ± 102<br>229 (148)	231 ± 113<br>229 (121)	p=0.388
**3α-AG (ng/mL)**	231 ± 173<br>158 (275)	200 ± 182<br>148 (148)	271 ± 171<br>201 (256)	393 ± 384<br>245 (412)	490 ± 516<br>347 (300)	p=0.032<br>0-9: p=0.028

						0-12: p=0.018
**Androstenedione (ng/mL)**	2.93 ± 1.07<br>2.80 (1.3)	2.96 ± 0.90<br>2.90 (1.3)	3.08 ± 1.00<br>3.05 (1.8)	3.47 ± 1.05<br>3.45 (2.7)	4.03 ± 1.36<br>4.10 (2.7)	*p<0.001<br>0-9: p=0.028<br>0-12: p=0.015
**Serum PSA (ng/mL)**	0.023 ± 0.015<br>0.02 (0.02)	0.032 ± 0.02<br>0.025 (0.02)	0.04 ± 0.03<br>0.03 (0.04)	0.041 ± 0.04<br>0.035 (0.03)	0.048 ± 0.03<br>0.040 (0.04)	*p<0.001<br>0-12: *p<0.001
**Urine PSA (ng/24 hours)**	71 ± 54<br>58 (54)	205 ± 470<br>85 (93)	270 ± 551<br>96 (183)	488 ± 795<br>248 (375)	568 ± 727<br>296 (510)	p=0.110

DHEA-S, Dehydroepiandrosterone sulfate; FSH, follicle-stimulating hormone; LH, luteinizing hormone; PSA, prostate-specific antigen; SHBG, sex hormone-binding globulin; 17-OHP, 17-Hydroxyprogesterone; 3α-AG, 3α-androstanediol glucuronide.

* p values correspond to the following: the upper p value is the result of the ANOVA test, and the other p values are those of the post hoc Scheffe test, but we are only presenting those that were statistically significant.
